# Effect of Layered Double Hydroxides on the Deterioration Process of Cement Paste under Sulfate Attack

**DOI:** 10.3390/ma15238437

**Published:** 2022-11-26

**Authors:** Lei Zhang, Linhua Jiang, Fangfang Zhi, Chunmeng Jiang, Weizhun Jin, Guohui Yang, Cheng Chen, Jianfeng Zhang

**Affiliations:** College of Mechanics and Materials, Hohai University, Nanjing 211100, China

**Keywords:** sulfate attack, layered double hydroxides (LDHs), cement paste, deterioration process, Vickers hardness, pore structure

## Abstract

This study investigated the effect of layered double hydroxides (LDHs) on the deterioration process of cement paste in the sulfate environment. Cement pastes with the addition of original and calcined LDHs at 2.5 wt.% and 5.0 wt.% of cement were exposed to Na_2_SO_4_ solution for 360 days. The macroscopic performance of the cement paste was assessed based on mass variation, porosity, compressive strength, and content of sulfate ions. Furthermore, the microhardness, microstructures, and composition of the degraded pastes were examined using Vickers hardness (HV), mercury intrusion porosimetry (MIP), scanning electron microscope (SEM), X-ray diffraction (XRD), and thermogravimetric analysis (TGA). The results indicate that cement paste incorporated with LDHs can mitigate the corrosion caused by sulfate effectively, especially in the case of calcined LDHs (C-LDHs), which primarily increase the adsorption of sulfate. Compared with the control specimen, the 180 d compressive strength loss ratio of specimens with 2.5 wt.% and 5.0 wt.% of C-LDHs decreased by 63.66% and 80.51%, respectively. Moreover, LDHs can reduce the amount of ettringite crystals, densify the microstructure, and refine the pore structure to mitigate the cement paste’s sulfate corrosion significantly. Compared with the control specimen, the 180 d harmful pore volume fraction of specimens laced with 2.5 wt.% and 5.0 wt.% C-LDHs decreased by 43.77% and 54.51%, respectively. In terms of the content of C-LDHs, an optimal content of C-LDHs could ensure the dominant effect of adsorption, while excessive C-LDHs could refine pores. In addition, Vickers hardness has an excellent correlation with compressive strength, which could precisely predict the compressive strength. Moreover, by combining the Vickers hardness distribution and content distribution of sulfate ions, the cross-section of the paste could be classified into four regions to evaluate the deterioration process accurately: the degraded zone, the strengthened zone, the invaded zone, and the intact zone.

## 1. Introduction

Sulfate attack is one of the main factors causing the expansion damage of cement-based materials, which significantly influences the durability and reliability of concrete structures. Expansion damage is attributed to reactions between free SO_4_^2−^ and hydration products, such as Ca(OH)_2_ or C-S-H [1,2,3]. Sulfate attack on cement-based materials is a complex physical chemistry process. The expansion mechanism could be concluded as a “diffusion–reaction–expansion” process, and its reaction types primarily contain ettringite, gypsum, magnesium sulfate attack, and thaumasite sulfate attack [4,5,6,7]. Up to now, extensive investigations have been conducted on the sulfate attack of cement-based materials, primarily in the field of expansion mechanisms, influential factors, assessment methods, and simulation models [8,9,10,11]. For instance, Müllauer et al. [5] and Yu et al. [6] revealed the expansion damage mechanisms of cement-based materials in the sulfate environment. Xiong et al. [12,13] and Jiang et al. [14] evaluated the influence of sulfate type, cation type, and fly ash on the deterioration process of cement paste exposed to sulfate. Qin et al. [15] assessed the effect of CO_2_ curing and low-temperature sulfate solution on the performance degradation of cement-based materials. According to interface-controlled crystal growth mechanisms, Gu et al. [16] modeled the sulfate expansion of cement-based materials. Zuo et al. [17] carried out numerical research on the expansive volume strain of concrete under sulfate attack.

Currently, the approaches to enhancing the sulfate resistance of cement-based materials are mainly surface coating, the addition of supplementary cementitious materials (SCMs), and chemical admixtures [18,19]. Surface coating could enhance the hydrophobicity of the cement matrix surface and impede the permeability of water and sulfate ions [20,21]. SCMs, such as slag, fly ash, metakaolin, and silica fume could mitigate sulfate corrosion by densifying the pore structure of cement-based materials and improving the hydrated paste properties [18,22,23,24]. Chemical admixtures improve the sulfate resistance by increasing the sulfate threshold value and decreasing the corrosion rate [25,26,27,28,29]. In addition, changing the curing method, adjusting the ambient temperature, and adding agricultural waste ashes can also significantly improve the sulfate resistance of cement-based materials [15,30]. Previous studies [5,6] suggest that the expansion damage of cement-based materials in the sulfate environment is essentially relevant to the ettringite crystals precipitation in small pores (10–50 nm), which could generate stresses until it exceeds the tensile strength of the cement matrix, and higher sulfate concentration results in higher stresses. Therefore, it is essential to use high-efficiency chemical admixtures to decrease the sulfate ions’ concentration below the critical level in the pore solution to enhance the sulfate resistance of the cementitious system.

**Figure 1:**
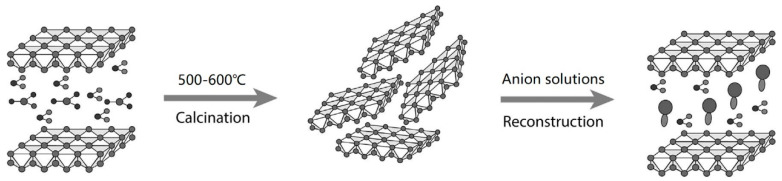
Schematic diagram of LDH structure reconstruction [34].

Layered double hydroxides (LDHs) are anionic layered compounds; the chemical formula is represented as ${\left[M_{1-x}^{2+}M_{x}^{3+}{\left(OH\right)}_{2}\right]}^{x+}{\left[A_{x/n}^{n-}\cdot mH_{2}O\right]}^{x-}$, where $M^{2+}$ and $M^{3+}$ are divalent and trivalent metal cations; $A^{n-}$ is the interlayer anions; x is the molar ratio of $M^{3+}/(M^{2+}+M^{3+})$. The layered structure is composed of metal hydrogen–oxygen octahedrons and the electronegative interlayer containing anions and water molecules linked by hydrogen bonds. Due to the weak hydrogen bond, the anions in the interlayers could be exchanged by other anions, which could intercalate into the interlayers more easily [31]. In addition, LDHs transform into layered double oxides (LDOs) after calcination, which can reconstruct the structure of LDHs in anion solutions by re-adsorbing anions, as shown in Figure 1. Therefore, the calcined LDHs tends to have excellent anion adsorption capacity [32,33]. 

Previous investigations primarily focus on the influence of LDHs on carbonation resistance and chloride binding; studies on the effect of LDHs on the sulfate resistance of cement-based materials are scarce and limited, especially those on the long-term sulfate attack deterioration process of cement-based materials. Chen et al. [35] evaluated the compressive strength and workability of concrete mixed with a combination of LDHs and metakaolin under drying–wetting cycles of sodium sulfate solution. Ma et al. [36] studied the effect of LDHs on the sulfate resistance of concrete specimens curing in a drying–wetting cycle environment by testing the expansion and compressive strength. Chen et al. [37] measured the volume variation of ultra-high-performance concrete (UHPC) specimens with the addition of LDHs in Na_2_SO_4_ solution. Guo et al. [31] assessed the sulfate resistance of concrete containing LDHs based on sulfate ion adsorption and compressive strength loss. The sulfate attack in an actual service environment is a long-term and complex physical chemistry process. However, few previous investigations have focused on the effect of LDHs on the long-term sulfate attack deterioration process of cement-based materials. Moreover, the evaluation methods are limited, mainly using traditional methods such as compressive strength loss or volume expansion, but compressive strength is an insensitive index and only evaluates the general mechanical behavior. Therefore, it is essential to use more-precise evaluation methods and figure out the impact of LDHs on the deterioration process of cement-based materials in the sulfate environment to predict the long-term strength and reliability of concrete structures.

The purpose of this research is to investigate the effect of LDHs on the deterioration process of cement paste in the sulfate environment. Moreover, the dominant effect of LDHs on sulfate resistance of cement paste was identified by evaluating the performance of sulfate resistance and the analysis of the composition, microstructure, and pore structure. The macro performance of cement pastes was evaluated based on mass variation, porosity, compressive strength, and content of sulfate ions. Furthermore, the microhardness, microstructures, and composition of the degraded pastes were examined using HV, MIP, SEM, XRD, and TGA.

## 2. Materials and Methods

### 2.1. Raw Materials and Mixture Proportions

**Figure 2:**
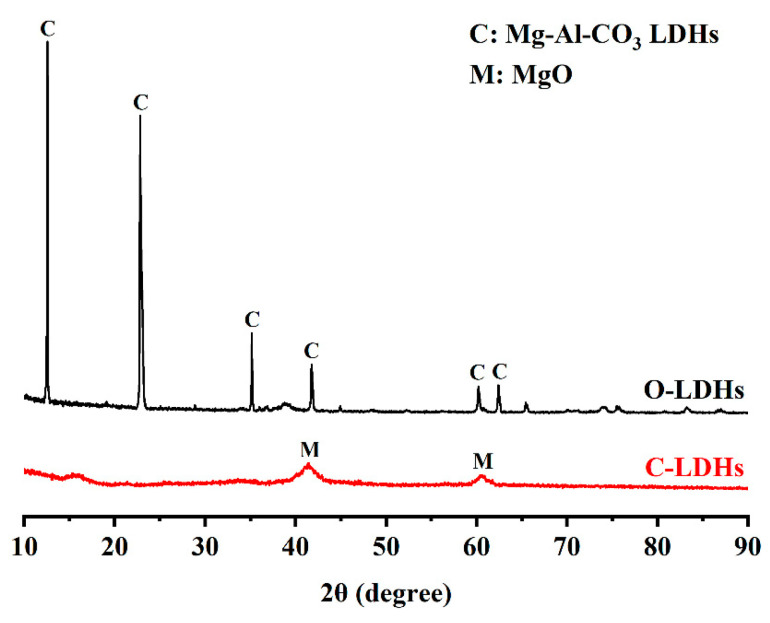
XRD patterns of O-LDHs and C-LDHs.

P. I 42.5 Portland cement (GB 175-2007 [38], namely ASTM C150 (2018) [39] Type I Portland cement) and Mg-Al layered double hydroxides (the molecular formula is Al_2_Mg_6_(OH)_16_CO_3_·4H_2_O) were used for this research. The chemical and mineralogical compositions of Portland cement and Mg-Al layered double hydroxides are presented in Table 1 and Table 2. The Mg-Al LDHs underwent two different preprocesses before the experiment: (I) drying at 105 °C for 24 h in a drying oven, marked as O-LDHs; (II) calcination at 550 °C for 6 h in a muffle furnace, marked as C-LDHs. The XRD patterns of O-LDHs and C-LDHs are shown in Figure 2. The main characteristic peaks of O-LDHs indicate well-crystallized Mg-Al-CO_3_ LDHs. However, the crystallinity of C-LDHs was weak, with a low intensity of MgO being detected. This phenomenon suggests that the layered structure of O-LDHs was destroyed and that the interlayer carbonate ions were almost removed after calcination.

Five different types of cement mixtures were prepared to assess the effect of LDHs on the sulfate resistance of cement paste, and the details of the cement paste mixture proportions are listed in Table 3.

### 2.2. Specimen Preparation and Corrosive Condition

**Figure 3:**
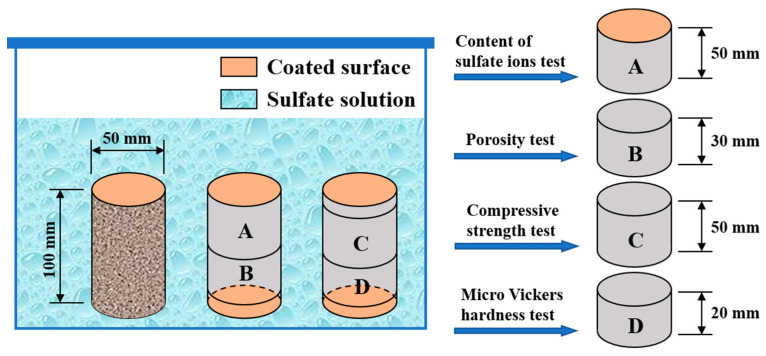
Schematic diagram of the experimental scheme.

According to Table 3, cylindrical specimens with 50 mm diameter and 100 mm height were cast and then cured in a standard environment (T = 20 ± 1 °C and RH = 95 ± 2%) for 24 h before demolding. All specimens were cured in saturated limewater at 20 ± 1 °C up to 91 d to minimize the interference of cement hydration on the experimental results and then divided into two groups equally. One group was immersed in Na_2_SO_4_ solution with a sulfate concentration of 5.0 wt.%. The bottom and top surfaces of the samples were coated with epoxy before the immersion to ensure that sulfate attack occurred on the side surface of the cylindrical specimens, and the exposure duration was 30, 90, 180, and 360 d, as shown in Figure 3. The other group of specimens remained in saturated limewater as a contrast. All specimens were stored in the laboratory at 20 ± 1 °C, and the Na_2_SO_4_ solution was renewed every month to keep the concentration constant. After immersion, the specimens were cut into small subsamples for content of sulfate ion, porosity, compressive strength, and Vickers hardness tests, as shown in Figure 3. In addition, intact cylinders were used for the mass variation test. Three specimens were prepared for every test, and the mean values were considered the final results.

### 2.3. Testing Methods

#### 2.3.1. Mass Variation and Porosity

Mass variation and porosity are the commonly used indicators to evaluate the cement paste’s sulfate corrosion degree. During this experiment, the mass variation (M) and porosity (P) of specimens were calculated as follows:

(1) $$M=\frac{M_{t}-M_{0}}{M_{0}}\times 100\%$$

(2) $$P=\frac{m_{t}-m_{0}}{V\rho_{w}}\times 100\%$$

where $M_{t}$ and $M_{0}$ are the mass of the same cylindrical specimens with and without sulfate exposure, respectively, which were air-dried to a constant value in the laboratory before measurement; $m_{t}$ is the saturated surface dry mass of subsample B (in Figure 3); $m_{0}$ is the mass of subsample B (in Figure 3) after drying at 40 °C for 24 h; V is the volume of subsample B (in Figure 3) measured using the drainage method; and $\rho_{w}$ is the water density [14].

#### 2.3.2. Compressive Strength

Compressive strength is considered the essential mechanical property to assess the deterioration degree of the cement paste under sulfate attack. During this study, the loss ratio of compressive strength (Δσ) was calculated as follows:

(3) $$\Delta \sigma =\frac{\sigma_{c}-\sigma_{d}}{\sigma_{c}}\times 100\%$$

where $\sigma_{c}$ and $\sigma_{d}$ are the compressive strength of subsample C (in Figure 3) in saturated limewater and 5.0 wt.% Na_2_SO_4_ solution, respectively.

#### 2.3.3. Vickers Hardness

**Figure 4:**
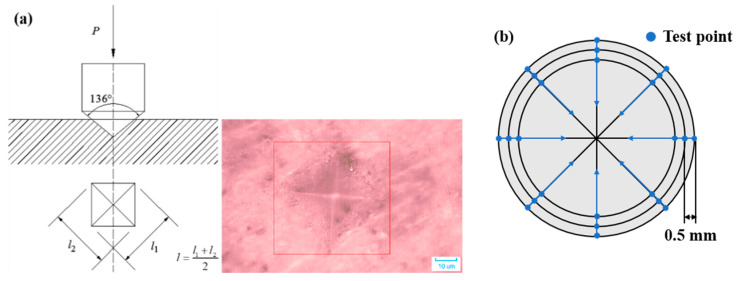
Schematic diagram (**a**) and the grid points (**b**) of Vickers hardness test.

Since the compressive strength is an insensitive index and only evaluates the general mechanical behavior, HV was applied to precisely assess the damage of all deteriorative positions in the cross-section [40]. Before the test, all the cross sections of subsample D (in Figure 3) were finely polished and dried at 40 °C for 24 h to minimize the humidity effect. HV was tested in steps of 0.5 mm from the external surface to the interior core. The measurement of HV was carried out with a load of 0.9807 N and then held for 15 s, as presented in Figure 4a. The HV was calculated as follows:

(4) $$HV=\frac{P}{A}=\frac{2P\sin\left(136^{\circ }/2\right)}{l^{2}}=\frac{1.854P}{l^{2}}$$

where P is the test load (kgf), A is the impression’s area (mm^2^), and l is the impression’s diagonal length (mm). The eventual value of HV was the mean of 8 test points of the same radius, as presented in Figure 4b.

#### 2.3.4. Content of Sulfate Ions

The content of sulfate ions is an essential indicator in evaluating the penetration and diffusion of the sulfate ions in the cementitious materials. Subsamples were prepared as shown in Figure 3 after 180 d immersion, then dried at 40 °C for 24 h in a drying oven. Finally, the cylinder subsamples were ground from the external surface to the interior core every 1 mm until 10 mm, and the powders were collected simultaneously. The content of sulfur was measured with an inductively coupled plasma emission spectrometer (Agilent 720ES). The sulfate ion content was calculated according to Equations (5) and (6):

(5) $$w_{so_{4}^{2-}}=\frac{3\times c_{s}}{10^{6}}\times 100\%$$

(6) $$c_{s}=\frac{c_{0}\cdot f\cdot V_{0}}{m_{0}}$$

where $c_{s}$ (mg/kg) is the content of sulfur; $c_{0}$ (mg/L) is the content of sulfur in the test solution, and the data are obtained by instrument testing; f takes the value 10, which means 10 times dilution; $V_{0}$ (mL) is the sample constant volume; $m_{0}$ (g) is the mass of the powder sample.

#### 2.3.5. XRD, TGA, SEM, and MIP

To further analyze the effect of LDHs on the hydration products and the microstructure of the cement pastes in the sulfate environment, typical samples (after 180 d immersion in 5.0 wt.% Na_2_SO_4_ solution) were detected by XRD, TGA, SEM, and MIP, respectively. XRD analysis was performed by a Rigaku Smartlab 9kw advance powder X-ray diffractometer with 1.54 Å Cu Ka radiation at a scanning speed of 10 °/min from 5° to 50°. TGA was carried out using a NETZSCH STA 449F3 Synchronous Thermal Analyzer at a heating speed of 10 °C/min from 30 to 800 °C in a flowing nitrogen atmosphere. The microscopic morphology of the samples was observed using a Zeiss Sigma 300 scanning electronic microscope. Before the test, the typical flaky samples were coated with a 10 nm thick gold film. MIP was tested using a Micromeritics Auto Pore V 9620 mercury porosimeter with a maximum pressure of 60,000 psi.

## 3. Results and Discussion

### 3.1. Mass Variation and Porosity

**Figure 5:**
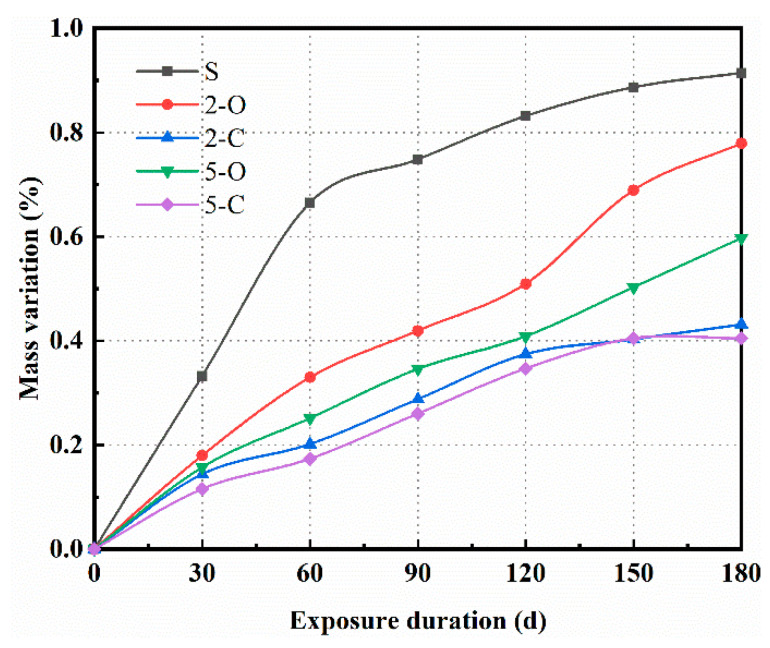
Mass variation of samples under sulfate attack.

Mass variation is a common indicator that reflects the quantitative variation of the specimen’s deterioration products in the sulfate environment. Figure 5 indicates the mass variation of five specimens exposed in 5.0 wt.% Na_2_SO_4_ solution. As expected, the mass variation of all specimens increased with increasing exposure duration. Obviously, the specimens incorporating LDHs underwent lower mass variation compared to the reference specimen at any exposure duration. It is noteworthy that C-LDH-incorporated specimens, 2-C and 5-C, displayed better sulfate resistance when exposed to Na_2_SO_4_ solution. This suggests that LDHs could enhance the sulfate resistance of cement paste, especially in terms of C-LDHs. In addition, it could follow that the calcination preprocessing is more important than the content for LDHs since the 2-C specimen performed better than the 5-O specimen.

**Figure 6:**
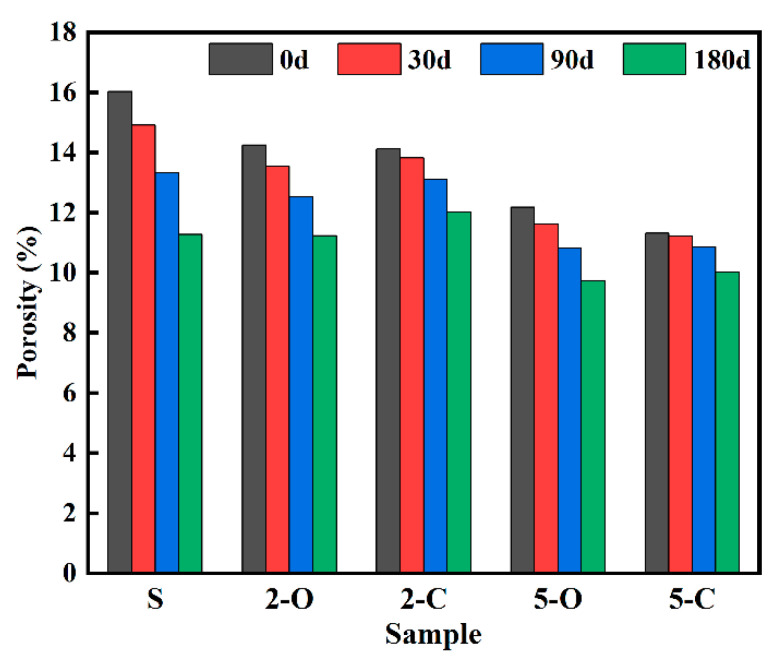
Porosity of samples under sulfate attack.

Since the porosity is closely associated with the permeability of sulfate ions, a water absorption test was performed to determine porosity in this study. The development of porosity for all samples exposed to Na_2_SO_4_ solution is presented in Figure 6. It was found that the porosity decreased with continuous exposure to Na_2_SO_4_ solution. At the same exposure duration, the porosity for the five samples decreased in the order: S, 2-O, 5-O, 2-C, and 5-C, which agrees well with the results obtained for mass variation.

### 3.2. Compressive Strength

**Figure 7:**
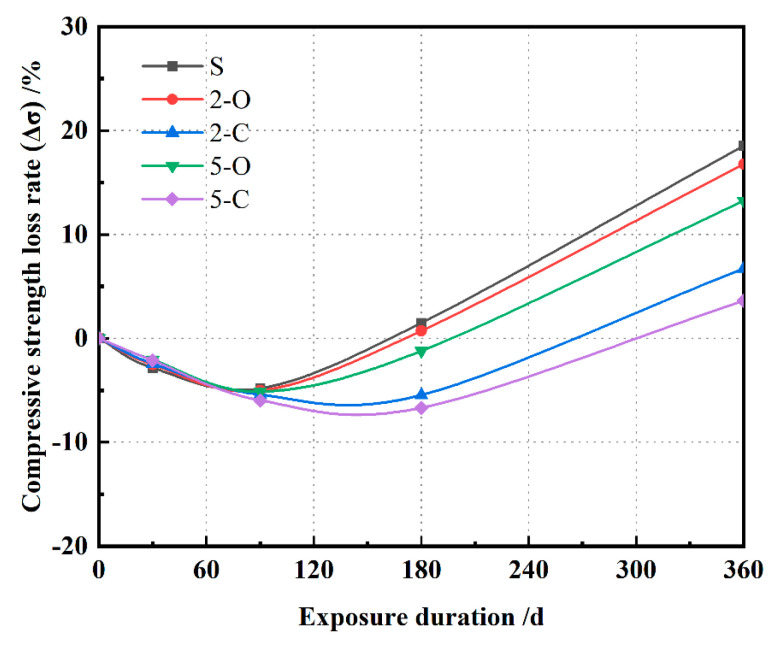
Compressive strength loss rates of samples under sulfate attack.

The loss ratio of compressive strength (Δσ) of the five specimens exposed to sulfate solution is presented in Figure 7. The result shows that Δσ for all specimens firstly decreased and then increased notably. For S, 2-O, and 5-O samples, the compressive strength increased in the first 90 exposure days, while it increased continuously in the first 180 days for 2-C and 5-C, which was owing to the further hydration, and the interior of the specimens was in intact condition [13]. In the first 90 days, the S specimen displayed slightly higher Δσ than the other four specimens. It demonstrates that the intact core played a pivotal role in the mechanical properties of the cement paste in the early exposure stage, and the surface corrosion had little effect on the compressive strength. In the later exposure stage, the compressive strength of S, 2-O, and 5-O started to come down, primarily due to the damage accumulation by chemical and physical sulfate attack, which reduced the intact core. The 2-C and 5-C specimens still maintained high compressive strength until 180 days and then began to fall. After 360 days, the compressive strength of the S, 2-O, 2-C, 5-O, and 5-C specimens was lost by 18.52%, 16.76%, 6.73%, 13.23%, and 3.61%, respectively, which proves that on adding LDHs cement paste has better sulfate resistance, especially when C-LDHs is added. Furthermore, C-LDHs can absorb a certain amount of free water, which leads to a lower w/b ratio and higher compressive strength [32].

Furthermore, the inhibition ratio (α) was calculated to better assess the influence of LDHs on the degradation of compressive strength under sulfate attack [41], as presented in Equation (7):

(7) $$\alpha =\frac{\Delta \sigma_{0}-\Delta \sigma }{\Delta \sigma_{0}}\times 100\%$$

where α (%) is the inhibition ratio of samples under sulfate attack, and $\Delta \sigma_{0}$ (%) is the compressive strength loss ratio of S specimen under sulfate attack.

**Figure 8:**
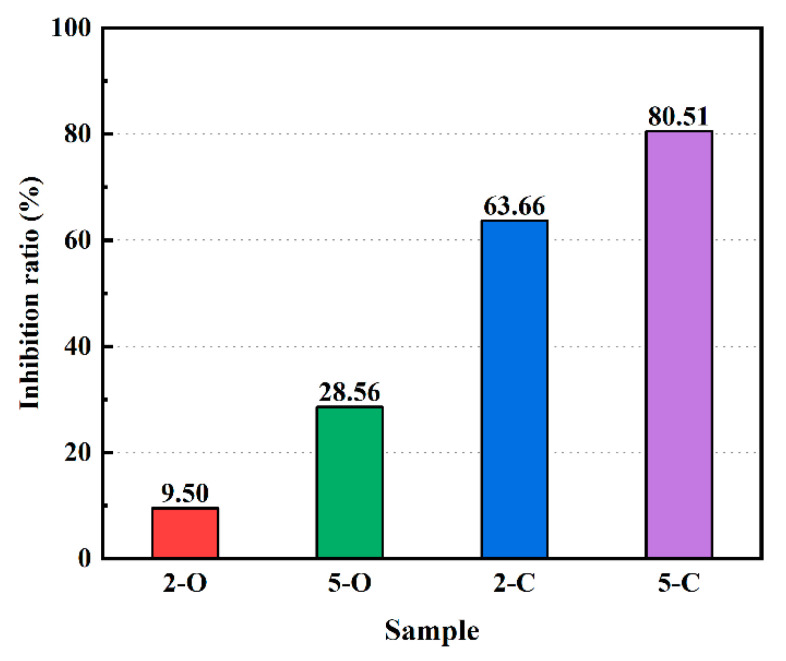
Inhibition ratios of samples under sulfate attack.

According to Equation (7), α is between 0 and 1 in the later period of exposure, and a higher α indicates that the sample exhibits better sulfate resistance. The inhibition ratio of the five specimens under sulfate attack after 180 d immersion was calculated and is presented in Figure 8.

Figure 8 illustrates that the order of the inhibition ratios of samples from large to small is 5-C, 2-C, 5-O, and 2-O, which is in line with the results obtained for compressive strength. The values of 2-C and 5-C specimens reached 63.66% and 80.51%, respectively, which are significantly higher than those for 2-O and 5-O. It confirms that adding C-LDHs into cement paste is an efficient approach to improving its sulfate resistance. However, the compressive strength may be an insensitive indicator since the sulfate attack on cementitious materials has a sluggish penetration and diffusion procedure from outside to inside. Therefore, some more effective assessment indicators should be proposed to assess the corrosion of sulfate attack.

### 3.3. Vickers Hardness

Since the compressive strength only evaluates the mechanical behavior of cement paste in general, the HV test was conducted to assess the mechanical behavior of all damaged areas [42]. HV is sensitive to the capillary and pore volumes and the Ca/Si, which reflect the microstructures and compositions of the tested specimens [13]. During this study, the HV of all the specimens under sulfate attack was tested.

**Figure 9:**
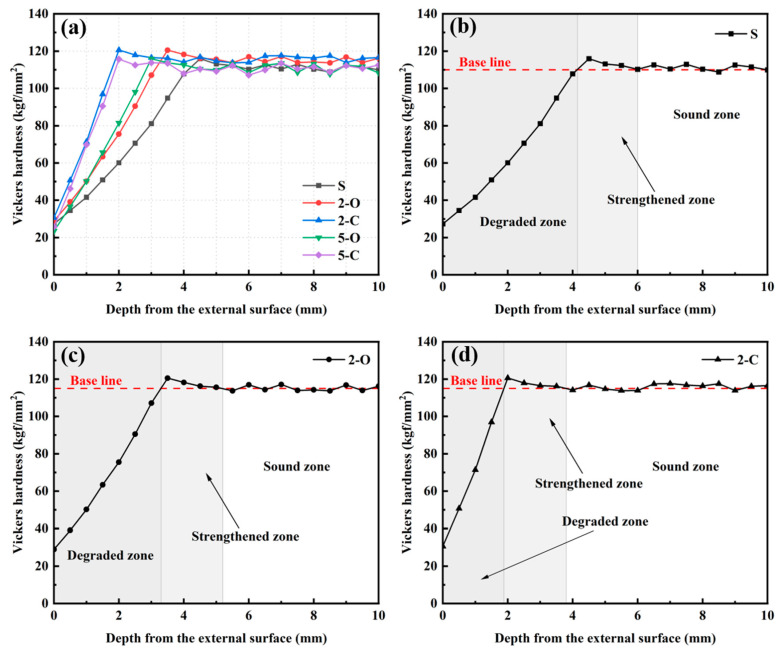
Vickers hardness of specimens under sulfate attack ((**a**): all specimens; (**b**): the S specimen; (**c**): the 2-O specimen; (**d**): the 2-C specimen).

Figure 9a presents the variation of HV with the depth from the external surface of specimens after 180 d immersion in Na_2_SO_4_ solution. There is a considerable difference between the HV of the exposed surface and that of the inner intact zone. When the depth reached a particular value, the values of HV became stable. It can be observed that the HV loss takes place faster on the S specimen than on other specimens. One reason is that LDHs decrease the volume and connectivity of capillary pores in the cement matrix, which can be inferred from the MIP results, thus leading to the densification of the microstructure and retardation of the mobility of SO_4_^2−^. Another more important reason is that LDHs could adsorb SO_4_^2−^ permeated into the specimens, and it would decrease the SO_4_^2−^ concentration in the pore solution that could react with hydration products, thus inhibiting cement expansion. The 2-C and 5-C samples performed better since C-LDHs have more active centers between the layers than O-LDHs, which could adsorb and exchange more SO_4_^2−^.

In addition, along the direction of the SO_4_^2−^ diffusion, the HV distribution on the cross-section was classified into three distinct regions (Figure 9b–d): the degraded zone, from the exposed surface to the position at which the HV rises to the baseline, where the HV dropped significantly, indicating that it had deteriorated due to sulfate attack; the sound zone, from the position the HV stabilizes at baseline to the core of specimens, where the HV remained stable, indicating that it had not been attacked by sulfate; and the strengthened zone, between the degraded zone and the sound zone, where the HV above the baseline and a peak value appears, indicating that it had been attacked and formed expansive compounds to fill the harmful pores [43]. As presented in Figure 9b–d, the depth of the degraded zone of the 2-C sample is the shallowest, less than half that of the S sample, which fully shows that the addition of C-LDHs effectively inhibits sulfate corrosion.

### 3.4. Relations between Vickers Hardness and Compressive Strength 

Compressive strength and HV are the two main parameters reflecting the mechanical properties of materials. Therefore, under certain conditions, a functional relationship between compressive strength and HV can also be established [12]. Since the compressive strength represents the mechanical behavior in general, the HV assesses the mechanical behavior of an individual point, and we need to define a new concept, equivalent Vickers hardness ($\bar{HV}$), namely the mean of HV, and calculated as in Equation (8):

(8) $$\bar{HV}\left(t\right)=\frac{2\pi \int_{0}^{r_{0}}HV\left(x,t\right)\cdot \left(r_{0}-x\right)\cdot dx}{\pi r_{0}^{2}}$$

where $\bar{HV}\left(t\right)$ means the equivalent Vickers hardness of specimens immersed for t days; HV(x,t) is the mean HV of eight test points of the same radius; x is the distance from the test point to the external surface; $r_{0}$ is the radius of cylindrical specimens.

**Figure 10:**
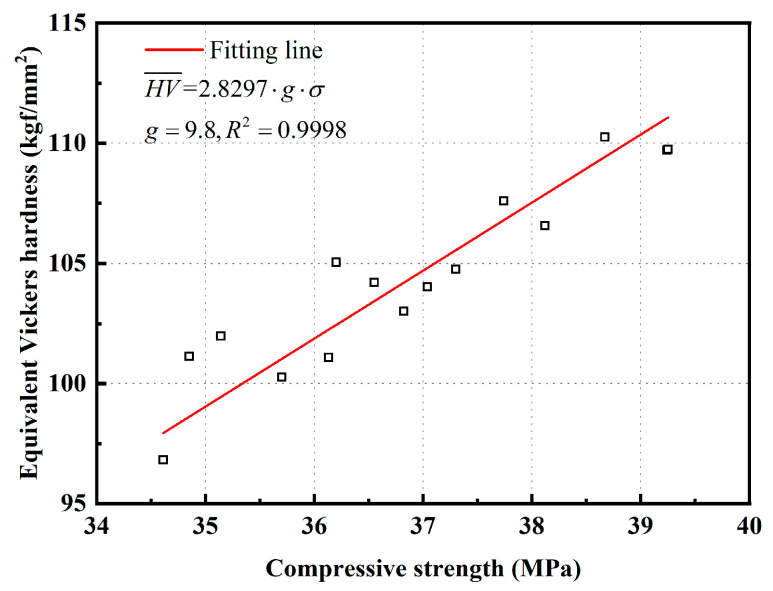
Relationship between $\bar{HV}$ and compressive strength.

According to Equation (8), the $\bar{HV}$ of specimens immersed in Na_2_SO_4_ solution for 30, 90, and 180 d was calculated, and the results are listed in Table 4. Moreover, the relationship between $\bar{HV}$ and compressive strength of specimens was fitted, as demonstrated in Figure 10. It can be observed that $\bar{HV}$ yields a strong linear relationship with the compressive strength, as shown in Equation (9), and the correlation coefficient (*R*^2^) is 0.998.

(9) $$\begin{array}{l} \bar{HV}=2.8297\cdot g\cdot \sigma \\ g=9.8 \end{array}$$

Figure 10 indicates that $\bar{HV}$ and compressive strength are excellently correlated, and therefore, $\bar{HV}$ is a valid criterion to assess the damage under sulfate attack. According to Equations (8) and (9), the compressive strength can be precisely predicted by testing the HV. Furthermore, a model of HV according to the logistic function could be established for long-term forecasting of the compressive strength [40].

### 3.5. Content of Sulfate Ions

**Figure 11:**
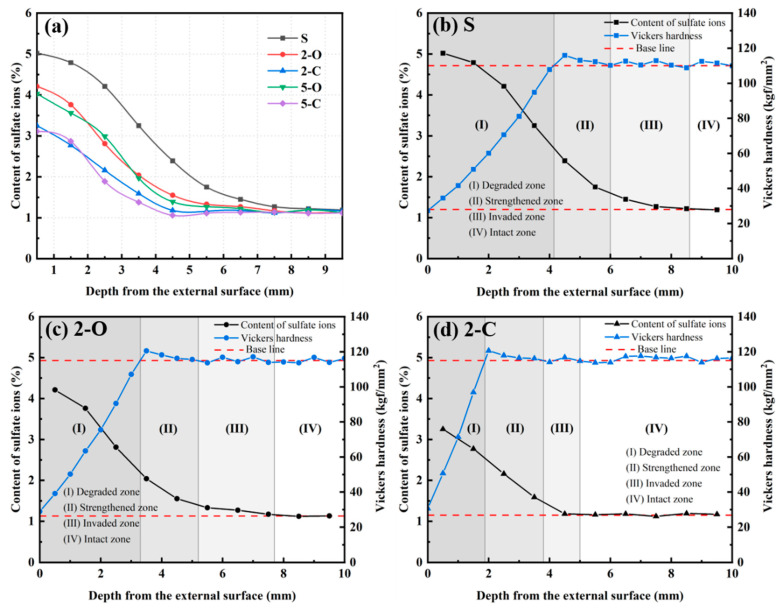
The SO_4_^2−^ content of cement paste under sulfate attack ((**a**): all specimens; (**b**): the S specimen; (**c**): the 2-O specimen; (**d**): the 2-C specimen).

The variation of the SO_4_^2−^ content with the distance from the exposed surface for samples after 180 d immersion in Na_2_SO_4_ solution is illustrated in Figure 11a. Since SO_4_^2−^ continuously diffuses from the external solution into the inner core of the cement matrix, the SO_4_^2^ content decreases with increasing depth. It is observed that the SO_4_^2−^ content of the exposed surface decreases in the sequence S > 2-O > 5-O > 2-C > 5-C. In addition, when the depth reached a particular value, the values of SO_4_^2−^ content almost remained constant, similar to the variation of the HV with the depth. It is reported that the corrosion depth under sulfate attack could be fixed by the variation of the SO_4_^2−^ content, from the exposed surface to the position with an almost constant SO_4_^2−^ content [14,44]. The sample with the largest corrosion depth was S, then followed by 2-O, 5-O, 2-C, and 5-C. This indicates that LDHs could inhibit sulfate corrosion by retarding the penetration of SO_4_^2−^.

As presented in Figure 11b–d, combining the HV distribution, the above-mentioned sound zone can be further divided into two regions: the invaded zone, where the SO_4_^2−^ content increases, but the HV is stable, and the intact zone, where the SO_4_^2−^ content and the HV are both stable. As seen in Figure 11, except for the core intact zone, the depths of the other three regions decrease in the order S, 2-O, and 2-C, which is in line with the results obtained for HV.

### 3.6. X-ray Diffraction (XRD)

**Figure 12:**
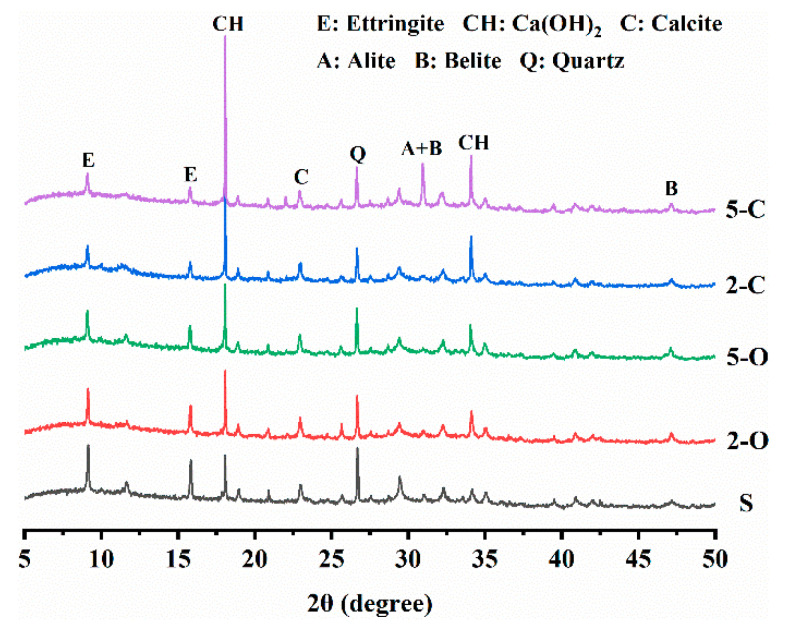
X-ray diffraction patterns of specimens under sulfate attack.

Figure 12 illustrates the XRD patterns of the five samples with immersion in Na_2_SO_4_ solution for 180 d. The strong peaks observed at about 9.2°, 15.8°, 18.1°, and 34.1° are identified as ettringite and calcium hydroxide, which are the major crystalline cement hydrates under sulfate attack [44,45]. The intensity of the ettringite diffraction peak decreases in the sequence S > 2-O > 5-O > 2-C > 5-C. Moreover, the intensity sequence of the calcium hydroxide peak is precisely the opposite of that of ettringite. It indicates that LDHs inhibit the formation of ettringite crystals, thereby decreasing stresses in small pores.

### 3.7. Thermogravimetric Analysis (TGA)

**Figure 13:**
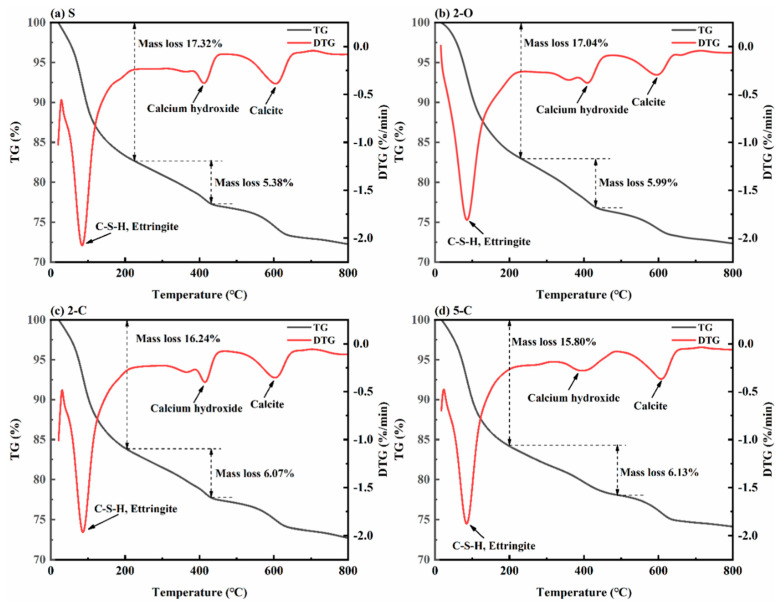
TG/DTG curves of specimens under sulfate attack ((**a**): the S specimen; (**b**): the 2-O specimen; (**c**): the 2-C specimen; (**d**): the 5-C specimen).

Figure 13 illustrates the TG/DTG curves of samples after exposure to Na_2_SO_4_ solution for 180 d. In Figure 13, the peaks observed at 80–100 °C are primarily owing to the dehydration of ettringite and C-S-H gel; the peaks appearing between 350 °C and 450 °C should be related to the dehydration of calcium hydroxide, and the peaks at 600–700 °C are linked to the decomposition of calcite [45,46,47]. It was found that the major corrosion product of all samples was ettringite, and calcium hydroxide was one of the primary reactants in the formation of ettringite. Based on the TG curves, the mass loss of ettringite and C-S-H was quantitatively calculated as 15.80% for the 5-C sample, which is less than that of the other samples. However, the mass loss of calcium hydroxide was exactly the opposite. This phenomenon is also found in the XRD patterns.

### 3.8. Scanning Electron Microscope (SEM)

**Figure 14:**
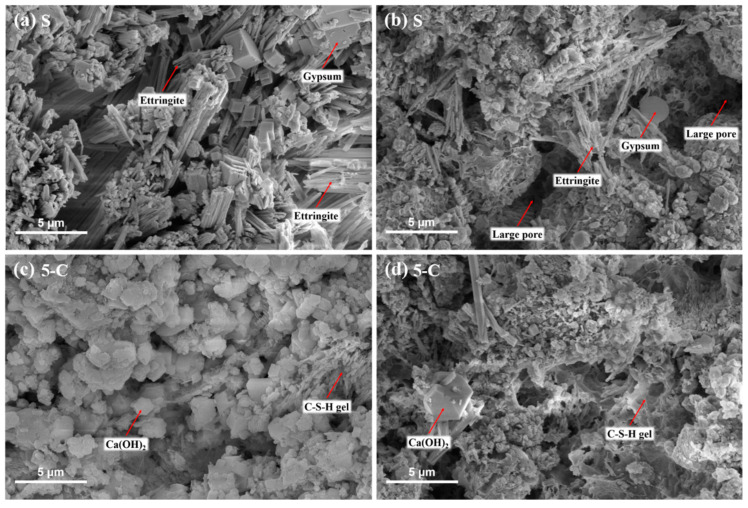
SEM micrographs of specimens under sulfate attack.

For the sake of examining the effects of LDHs on the major corrosion products under sulfate attack, SEM analysis was conducted on the specimens after 180 d immersion. Figure 14a illustrates that needle-shaped ettringite and plate-like gypsum crystals were embedded in the deteriorated S sample, and few C-S-H gels and portlandite crystals were observed. The expansive products deposited in the pores of samples led to increased mass variation and decreased porosity. It could also account for the phenomenon of the “strengthened zone” in the Vickers hardness distribution. With continuous exposure to the sulfate solution, more and more ettringite crystals formed and precipitated in the small pores, leading to the formation of large pores and cracks in cement matrix (Figure 14b). It confirms that the deterioration of the cement pastes under sulfate attack is mainly due to the formation and precipitation of ettringite crystals in small pores. Compared with the S sample, 5-C exhibits a denser microstructure with few ettringite and gypsum crystals as observed in Figure 14c. Moreover, a certain amount of C-S-H gel and portlandite crystals were observed in the 5-C sample (Figure 14d). It indicates that 5.0 wt.% C-LDHs could effectively improve the sulfate resistance of cement paste, which is related to the denser microstructure and lower sulfate content in the cement paste. This is attributed to the filling effect of C-LDHs in large pores and the adsorption effect of C-LDHs on sulfate ions in the pore solution.

### 3.9. Mercury Intrusion Porosimetry (MIP)

**Figure 15:**
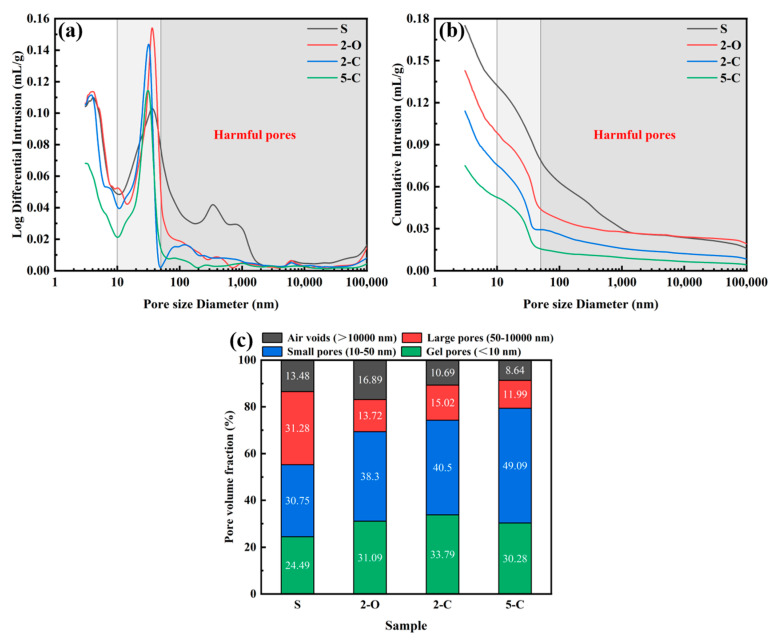
Pore structure characteristics of specimens under sulfate attack.

The pore structure characteristics of samples after 180 d immersion in Na_2_SO_4_ solution are presented in Figure 15. Figure 15a shows that adding LDHs shifts the distribution curves toward the direction of smaller pore size. Additionally, the most probable pore diameter (corresponding to the peak of the differential pore size distribution curve) decreases with the addition of LDHs. According to previous research [48,49,50], the pores in the cement matrix could be classified into three types: large pores (harmful pores) with sizes more than 50 nm, small pores (less-harmful pores) with sizes between 10 and 50 nm, and gel pores (harmless pores) with sizes less than 10i3 nm. The expansion damage of cementitious materials under sulfate attack is relevant to the precipitation of ettringite crystals in small pores. Figure 15b illustrates that the harmful pore volume remarkably reduces after incorporating LDHs, and the decrease sequence is as follows 5-C > 2-C > 2-O. Figure 15c demonstrates that adding LDHs refines the pore structure by dropping the volume fraction of harmful pores.

In addition, it is worth noting that the proportion of large pores for 2-C and 2-O samples are almost identical. According to the above experimental results, the 2-C sample performed much better resistance to sulfate attack than the 2-O sample. This can probably be attributed to the more robust adsorption capacity of C-LDHs. Thence, it can be preliminarily judged that the adsorption effect is dominant when the C-LDHs content is 2.5 wt.%. On the other hand, the 2-C sample performed merely a little worse than the 5-C sample on sulfate resistance. However, the proportion of large pores of 5-C is further decreased. It is most probable that the SO_4_^2−^ concentration in the pore solution has already decreased below the critical level when the C-LDHs content is 2.5 wt.%, which inhibits the formation and precipitation of ettringite crystals in small pores of C-S-H gels [6]. The excessive C-LDHs mainly fill the large pores and reduce the water to refine the pore structure. In addition, for uncalcined O-LDHs, both the adsorption effect and refine effect played a major role since the SO_4_^2−^ concentration in the pore solution had not yet fallen below the critical level.

## 4. Conclusions

This research was performed to figure out the effect of LDHs on the deterioration process of cement paste under sulfate attack. The following conclusions can be drawn based on the above results:

(1) Cement pastes bolstered with LDHs have excellent adsorption capacity for sulfate ions. Adding LDHs can mitigate the corrosion of sulfate effectively. Calcination treatment could result in more active sites between the layers of LDHs, which can adsorb and exchange more sulfate ions and further improve the adsorption capacity for sulfate ions. For LDHs, calcination is more important than the content.
(2) The LDHs’ adsorption of sulfate ions from the pore solution reduces the amount of expansion products, such as ettringite crystals, which decreases the stress in small pores and mitigates the expansion damage of cement paste. Furthermore, LDHs densifies the microstructure by refining the pore structure, thus reducing the sulfate diffusion in cement paste.
(3) Based on this experiment’s results, 2.5 wt.% is an optimal content for calcined LDHs since the adsorption effect is dominant when the content is 2.5 wt.%; excessive addition of C-LDHs primarily plays a role in refining pores, and the ion adsorption and exchange capability cannot be fully realized. Furthermore, LDHs are not cementitious materials, and excessive LDHs could have an inhibitory effect on the cement hydration, which impacts the development of compressive strength.
(4) Vickers hardness is a valid criterion to assess the deterioration process of cement paste under sulfate attack, which takes into account the average and the individual. The equivalent Vickers hardness is proposed to evaluate the mechanical behavior in general, and it has an excellent correlation with the compressive strength. Consequently, the compressive strength can be precisely predicted by testing the HV. Furthermore, the cross-section of the paste could be classified into four distinct regions by combining the HV distribution and SO_4_^2−^ content distribution, namely the degraded zone, the strengthened zone, the invaded zone, and the intact zone, to evaluate the deterioration process accurately.

## Figures and Tables

**Table 1:** Chemical and mineralogical compositions of Portland cement (wt.%).

Composition	CaO	SiO_2_	Al_2_O_3_	Fe_2_O_3_	MgO	SO_3_	R_2_O	C_3_S	C_2_S	C_3_A	C_4_AF
	62.83	20.50	5.61	3.84	1.70	3.07	1.05	48.0	22.9	8.4	11.7

**Table 2:** Chemical and mineralogical compositions of Mg-Al layered double hydroxides (wt.%).

Composition	SiO_2_	MgO	Al_2_O_3_	Fe_2_O_3_	CaO	SO_3_	L.O.I.
O-LDHs	0.02	34.53	20.68	0.03	0.07	1.82	42.08
C-LDHs	0.01	55.92	34.87	0.03	0.12	2.98	5.32

**Table 3:** Mixture proportions of cement paste.

Specimen	O-LDHs/%	C-LDHs/%	PC/%	w/b
S	0	0	100	0.4
2-O	2.5	0	100	0.4
2-C	0	2.5	100	0.4
5-O	5.0	0	100	0.4
5-C	0	5.0	100	0.4

**Table 4:** Equivalent Vickers hardness and compressive strength of specimens under sulfate attack.

Exposure Duration (d)	Specimen	$\bar{HV}$ (kgf/mm^2^)	Compressive Strength (MPa)
30	S	101.08	36.13
	2-O	107.60	37.74
	2-C	106.58	38.12
	5-O	101.97	35.14
	5-C	100.27	35.70
90	S	103.01	36.82
	2-O	110.25	38.67
	2-C	109.71	39.24
	5-O	105.05	36.20
	5-C	104.03	37.04
180	S	96.83	34.61
	2-O	104.21	36.55
	2-C	109.74	39.25
	5-O	101.13	34.85
	5-C	104.76	37.30

## Data Availability

The data presented in this study are available on request from the corresponding author.
